# A Review of Pore Water Pressure Measurement Techniques in Early-Age Cement-Based Materials

**DOI:** 10.3390/ma18163875

**Published:** 2025-08-19

**Authors:** Qian Tian, Yang Wang, Hua Li, Yujiang Wang, Chen Jiang

**Affiliations:** 1 State Key Laboratory of Engineering Materials for Major Infrastructure, Jiangsu Sobute New Materials Co., Ltd., Nanjing 211103, China; wangyang@cnjsjk.cn (Y.W.); lihua@cnjsjk.cn (H.L.); 2 Jiangsu Key Laboratory of Construction Materials, College of Materials Science and Engineering, Southeast University, Nanjing 211189, China; yjwangjs@seu.edu.cn; 3 Jiangsu Provincial Communications Construction Bureau, Nanjing 210004, China

**Keywords:** cement-based materials, porous media, early age, pore water pressure, testing methodology

## Abstract

The evolution of early-age structure in fresh cement-based materials fundamentally involves a transition from a suspended dispersion system to a porous medium, accompanied by changes in the energy state of the internal water. Monitoring pore water pressure (PWP) evolution reflects these changes in water energy state and provides insight into the underlying mechanisms governing the development of early-age performance in cement-based materials. Building on concepts from soil physics, this paper examines the thermodynamic mechanisms driving PWP evolution during the early stages of cement-based materials’ formation. It further synthesizes advances in PWP testing methodologies and instrumentation for cement-based materials, alongside their applications in both fundamental research and engineering practice.

## 1. Introduction

Water is an essential constituent of cement-based materials (cement paste, mortar, and concrete), playing a decisive role in their performance development. Prior to setting, water acts as a dispersion medium within the suspended particle system, facilitating particle lubrication and dispersion while governing workability [1]. Exposure to drying conditions at this stage induces water evaporation, influencing plastic shrinkage cracking behavior [2,3,4,5,6]. Throughout setting and hardening, water enables hydration reactions, being progressively consumed to form hydration products. Residual water occupies pores within the resulting porous medium, subsequently affecting mechanical properties and durability [7]. Water consumption and evaporation govern autogenous and drying shrinkage characteristics [8,9].

During distinct hydration stages, water exists in varied forms corresponding to specific energy states within cement-based materials. For example, the evaporation of fresh cement-based materials can be divided into three stages according to the thermodynamic state of the evaporated water [1,5]. The decrease of internal relative humidity is directly correlated to the autogenous shrinkage of cement-based materials [10,11]. Monitoring these energy state changes is critical for understanding the performances evolution of these kinds of materials, from many aspects. Particularly during the early-age period from mixing through initial hardening, systems remain highly saturated (relative humidity ≈ 98–100%) despite ongoing water consumption and evaporation [11]. This near-saturated state complicates moisture characterization in a super-hygroscopic regime (typically >95% RH), presenting significant scientific and technical challenges for conventional hygrometer measuring techniques due to complex phase behaviors, material interactions, and instrumentation limitations [12,13]. Directly measuring the pore water pressure (PWP) of early-age cement-based materials addresses the limitations of conventional hygrometers and sensitively captures the evolution of internal water energy during the super-hygroscopic stage, whether under sealed or evaporating conditions. This provides crucial characterization of the driving force behind plastic and autogenous shrinkage cracking, which are the primary causes of early-age cracking and service life degradation in modern concrete [14,15,16]. Moreover, during this stage, cement-based materials undergo an abrupt transition from a suspended dispersion system to a porous medium, marked by inflection points in many key properties [17,18,19]. The accurate determination of this transition time is the base for cracking stress calculation, time of surface finishing determination, appropriate curing procedures, etc. Measuring PWP also provides promising application prospects in this area. Therefore, PWP measurement of early-age cement-based materials with precise thermodynamic tracking of water evolution during this phase is essential for quality control and assuring the service life of modern concrete.

Over decades of development, PWP measurement has garnered increasing attention in cement-based materials research and engineering. Previous studies have validated its unique value and demonstrated engineering potential. However, no standardized framework yet governs this methodology. Substantial discrepancies in instrumentation configurations and measurement protocols across the literature have yielded inconsistent test outcomes and divergent interpretations, impeding reliable implementation of this technique. Based on thermodynamic analysis of pore water evolution in early-age cement-based materials, this paper delineates the historical evolution and contemporary advances in PWP measurement for early-age cement-based materials by examining the relevant published research papers, technical reports, patents, and doctoral theses, providing a systematic comparative analysis of existing methods. Evaluated parameters encompass operational principles, probe configurations, and measuring range; implementation approaches including saturation procedure, orientation and embedding depth of probes, repeatability, temperature influence, and filed adaptability. Additionally, this article synthesizes diverse applications of this kind of testing methodology in theoretical investigations and practical engineering implementations for cement-based materials. The synthesis supports future initiatives to standardize these measuring techniques in order to enhance quality assurance and the performance of modern cement-based materials.

## 2. Thermodynamic Evolution of Pore Water in Early-Age Cement-Based Materials

Similar to water in natural systems, moisture within water-mixed cement-based materials exists in multiple phases with distinct energy levels. Interactions such as adsorptive forces and capillary tension cause the energy state of this pore water to differ fundamentally from that of pure free water. In order to demonstrate the thermodynamic evolution of pore water in early-age cement-based materials, the concept of water potential in soil physics is referenced [20], defined as the work required to reversibly transport a unit quantity of water from cement-based material to a reference pool of pure free water at identical temperature and standard atmospheric pressure. Here, pure denotes solute absence, while free implies freedom from physical constraints. It should be noted that, unlike soil, early-age cement-based materials may undergo dramatic performance change. However, at an infinitesimal time interval during the hydration process, cement-based materials can be regarded as being in a thermodynamic quasi-static process. Water potential is governed by thermodynamic state parameters including temperature, pressure, solute concentration, and confinement intensity; it can be expressed under isothermal conditions through a modified Gibbs equation, as follows:

(1) $$\;\Psi_{w}={\left(\frac{\delta \Psi_{w}}{\delta P}\right)}_{T,\;n_{w},n_{j}}\Delta P+{\left(\frac{\delta \Psi_{w}}{\delta n_{w}}\right)}_{T,\;P,n_{j}}\Delta n_{w}+\sum_{j}{\left(\frac{\delta \Psi_{w}}{\delta n_{j}}\right)}_{T,\;p,n_{w}}\Delta n_{j}+\rho_{w}gz$$

where *ψ* denotes the total water potential in the cement-based material; *T*, *P*, *n_w_*, and *n_j_* represent temperature, pressure, water content, and the concentration of the *j*th solute, respectively; $\rho_{w}$ is the density of water; *g* is the gravitational acceleration; and z is the vertical distance from the reference position. The first term on the right side represents the pressure potential, which quantifies the work required to reversibly transport a unit quantity of water from a cement-based material to a system at the reference pressure (e.g., atmospheric pressure) while keeping all other states unchanged. If atmospheric pressure is chosen as the reference, the total pressure of water within the cement paste equals atmospheric pressure due to the connectivity between internal pores and the external environment. Consequently, no pressure potential exists in this case. The second term on the right side is the matric potential, also known as matric suction (S). It describes the reversible work required to move a unit quantity of water from the cement-based material to an environment with zero matric potential (pure free water unaffected by any physical constraints), under otherwise identical conditions. Matric potential originates from the adsorptive forces exerted by cement pore walls on water molecules and capillary tension. Numerically, it equals the difference between pore air pressure (U_a_) and pore water pressure (U_w_). Since the work capacity of water in cement pores is reduced by interactions with pore walls, matric potential is always negative. The third term on the right side is the solute potential, which represents the work required to transfer a unit quantity of water from the cement-based material to a solute-free system with identical conditions. Solute molecules adsorb water molecules, reducing water activity and lowering its free energy. As a result, the solute potential is always negative too, reflecting the diminished work capacity of the solution compared with pure water. The fourth term on the right side is the gravitational potential, defined as the work required to move a unit quantity of water vertically from its position in the cement-based system to a reference elevation, under otherwise identical conditions. Water potential (ψ) reflects changes in the Gibbs free energy of pore water within cement-based materials due to internal (adsorption/capillarity) and external (gravity) force fields. The energy evolution of water during hydration and hardening processes can thus be characterized by water potential dynamics via Equation (1).

Upon water mixing, cement-based materials initially form a suspension system consisting of solid particles of various sizes suspended in water. During this stage, ions such as K^+^, Na^+^, Ca^2+^, SO_4_^2−^ dissolve from unreacted cement grains [21], generating solute potential (ψ_s_) that reduces total water potential. The solid particles exert a gravitational force on the water, creating hydrostatic pressure and pressure potential (ψ_p_). At this stage, solid particles settle while water migrates upward, causing sedimentation and bleeding. When exposed to drying conditions, menisci form within the system once surface evaporation exceeds the bleeding rate. This develops matric potential (ψ_m_), further reducing water potential. As evaporation progresses inward and from larger to smaller pores, suction intensifies and water potential declines. Under sealed conditions without water evaporation, sedimentation of solid particles and the growth and gradual interconnection of hydration products form a percolating solid network within cement-based materials [22]. This structural framework can support its own weight, minimizing gravitational potential. Further hydration within this solid structure also forms menisci, generates matric potential and continuously lowers water potential through ongoing hydration.

Therefore, the energy state of the water in cement-based material evolves continuously after mixing, governed by hydration progression and environmental interactions. This dynamic process manifests as measurable water potential changes. For example, under sealed conditions, the measured value of pore water pressure of fluid cement-based materials immediately after placing equals the hydrostatic pressure of the concrete. Then, the pressure decreases with time as result of the formation of a “self-supporting” body [23]. Once the structural skeleton developed, a further decrease in internal pore water pressure induces a self-desiccation effect, which correlates directly to the autogenous shrinkage of cement-based materials. Under evaporated conditions, surface moisture evaporation generates suction pressure, which directly correlates to the plastic shrinkage and drying shrinkage of cement-based materials. Real-time monitoring of these potential variations is essential for understanding the underlying mechanisms of the early-age performance evolution of cement-based materials.

## 3. Development of Testing Methods and Instrumentation of Early-Age PWP for Cement-Based Materials

In 1976, Wittmann [24] pioneered the first experimental measurement of early-age PWP in cement-based materials by embedding a water-filled plastic tube equipped with a pressure sensor at its top into fresh concrete. Radocea [25,26,27,28] subsequently adopted similar instrumentation in 1991 to investigate the mechanism of plastic shrinkage for cement-based materials. Subsequent studies by Scott et al. (1997), Hammer et al. (2001), Holt et al. (2004), and Slowik et al. (2008) all employed analogous methods and apparatus for their research [23,29,30,31,32,33,34,35,36,37]. In 2006, Liu and Tian et al. developed an automated PWP testing system for early-age cement-based materials featuring a calcined porous ceramic head as the probe [38,39,40,41,42,43,44,45,46,47,48]. Ghourchian et al. conducted studies on plastic shrinkage cracking using similar equipment and methods [49,50]. Recently, Jamali et al. (2022) from Northumbria University, UK, developed an automated PWP testing system for early-age cement-based materials using a high-capacity tensiometer (HCT-C) they developed, capable of measuring elevated PWP values [51,52,53]. Concurrently, Deysel et al. utilized a high-capacity tensiometer to explore techniques for inhibiting early-age shrinkage cracking in concrete [54,55]. Table 1 summarizes and compares the detailed features of the instrumentations and methodological approaches across these studies.

### 3.1. Fundamental Principles of Measurement

**Figure 1:**
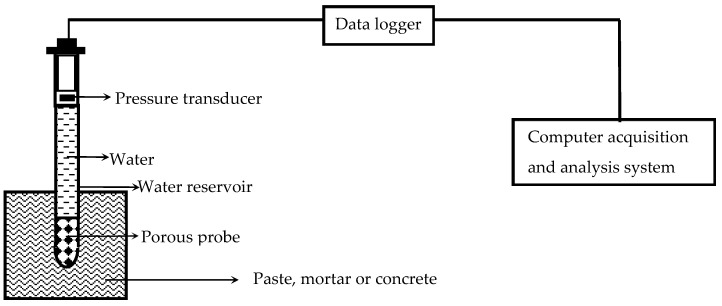
Measurement Principle of Early-Age PWP in Cement-based materials.

Although the instruments in Table 1 vary in design, the core measurement principles remain consistent with soil tensiometers. This approach employs a water-saturated probe with a porous tip. After concrete placement, this probe is immediately embedded into cement-based materials at specified depths. As shown in Figure 1, the probe’s pressure sensor at its rear end connects to a multi-channel data acquisition system, enabling automated monitoring of early-age PWP in cement-based materials. This methodology relies on the thermodynamic equilibrium between the probe’s internal water and cementitious matrix pore water. Surface tension creates contractile skin (menisci) at the porous interface of the pre-saturated probe. Within defined pressure ranges, this interface functions as a semi-permeable membrane, permitting free passage of water molecules and dissolved ions while blocking gas transmission. When the water-saturated probe is inserted into fresh cement-based materials, hydraulic continuity is established between the probe and cement-based materials, achieving initial equilibrium where water potentials are equal. The initial PWP measured immediately after placement reflects the hydrostatic pressure.

As cement hydration advances or environmental drying occurs, the cement-based material transitions from saturated to unsaturated state, forming a meniscus that reduces water potential. This gradient drives water flux from the tensiometer (higher potential) to the cement-based material (lower potential) through the porous probe until thermodynamic equilibrium is reestablished. Within this sealed system, the process generates measurable negative pressure relative to atmospheric reference.

The pressure transducer records gauge pressure—the differential between absolute internal pressure and atmospheric pressure—when ambient air pressure equals atmospheric pressure. Solute potential equilibrates rapidly between the probe and matrix water as dissolved ions can permeate freely through the semi-permeable membrane. Consequently, the measured pressure directly quantifies the matric suction (negative pore pressure) within the cement-based material, being numerically equivalent but opposite in sign.

### 3.2. Probe Design and Measurement Range

The measurable PWP range in tensiometers is fundamentally constrained by the measurement principle. As a sealed pressure measurement system, all components, including the porous probe, transducer, and encapsulated water reservoir, must be airtight and constructed from materials resistant to pressure-induced deformation and the alkaline environment in cement-based materials (e.g., ceramic tips, syringe needles, steel conduits, rigid polymers—see Table 1). Under the premise of enough airtightness, the primary metrological challenges involve gas-phase (permeated externally or generated internally) interference prevention. System reliability and measurement range are predominantly governed by air exclusion efficacy. Two critical range-limiting phenomena are as follows:

- Probe air-ingress: Gas permeation through porous interfaces.
- Cavitation-induced nucleation: Bubble formation in the water reservoir.

Hydrostatic pressure measurement (positive domain) governed by fluid statics (P = ρgh) can be measured using standard positive-pressure sensors. The negative pore pressure (suction) measurement faces thermodynamic constraints and is quite difficult to break through a theoretical limit. Methodological advancements consequently focus on increasing the peak value of PWP measurement.

As established in Section 3.1, tensiometer operation fundamentally relies on hydraulic continuity between the cementitious matrix’s internal moisture and the instrument’s water reservoir, mediated by the probe’s porous structure. This configuration enables liquid-phase transport while impeding gas-phase penetration. The water-saturated pore network within the ceramic probe functions as a semi-permeable membrane, exhibiting selective permeability governed by capillary principles. Within its operational envelope, this membrane permits unrestricted transport of water and dissolved ions while blocking air phase transmission.

The sustainable negative pressure range is thermodynamically constrained by the maximum air-entry pressure (*P*_*ae*_) of the probe material, determined by the Young-Laplace equation, as follows:

(2) $$P_{ae}=\frac{2\gamma cos\theta }{r_{max}}$$

where *r*_max_ denotes the radius of the largest continuous pore throat in the ceramic microstructure, *γ* the liquid-air surface tension, and *θ* the contact angle.

**Figure 2:**
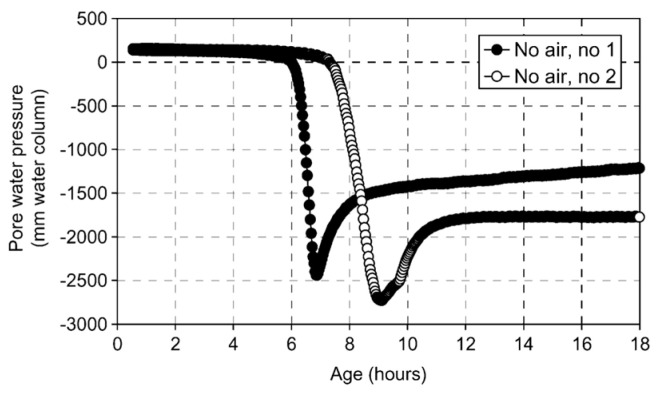
Inflection point in the PWP curve [30].

**Figure 3:**
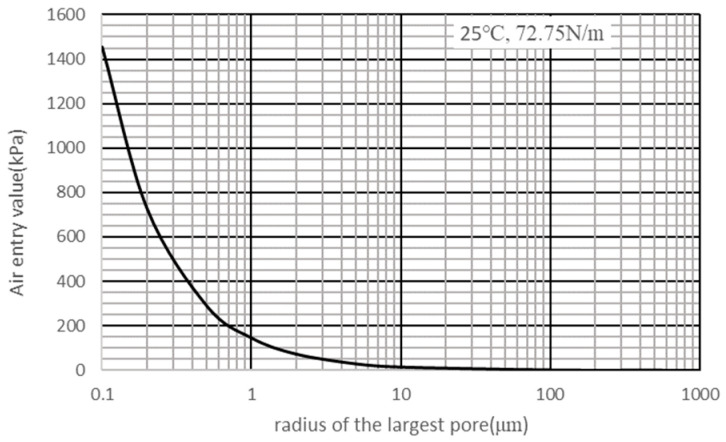
Development of AEV with pore radius.

This critical threshold pressure is termed the probe’s air-entry value (AEV), defined as the minimum pressure differential required for a non-wetting fluid phase (here, air) to displace water and penetrate the saturated porous matrix of the probe. It reflects the probe’s initial resistance to air intrusion. As expressed by Equation (2), the AEV exhibits an inverse relationship with pore radius; smaller maximum pore dimensions yield higher AEVs. When the absolute magnitude of measured negative pressure remains below the AEV, air penetration is thermodynamically inhibited, while liquid water and dissolved ions permeate freely through the capillary membrane, ensuring hydraulic continuity and enabling valid measurements. When the absolute magnitude of measured negative pressure exceeds the AEV, air menisci propagate through interconnected pore throats, the semi-permeable function collapses, and the hydraulic continuity is irreversibly compromised. This failure manifests experimentally as a sudden pressure decay in PWP-time data series and marked by characteristic inflection points (Figure 2). Consequently, the operational range of the tensiometer is dictated by the largest continuous pore throat in the ceramic microstructure. Figure 3 demonstrates that maximum sustainable negative pressure scales inversely with maximum pore size. To achieve measurable suctions in the kilopascal range (kPa), the probe’s maximum pore diameter must be reduced to the micrometer scale (μm).

**Figure 4:**
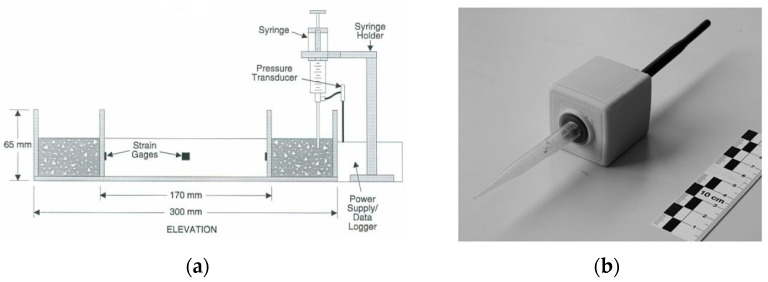
Probes using water-filled tube. (**a**) Syringe probe [31]; (**b**) plastic tube probe [33].

According to the probe configuration and the corresponding measurement range, the testing instrumentations listed in Table 1 can be classified into three categories, as follows:

(1) Tensiometers with tube probes. Early tensiometer probes developed by Wittmann and later researchers (Radocea [27], Scott et al. [31], Hammer [29], Slowik et al. [33].) share fundamental similarities. These designs typically employ water-filled systems comprising plastic tubing, syringes, or thin-wall metal conduits with orifice diameters of 1–3 mm (Figure 4). Wittmann’s configuration uniquely incorporated paper plug seals at plastic tube orifices. Given these dimensions, the theoretical air-entry value (AEV) calculated via Equation (2) at 20 °C ranges from 0.096 to 0.288 kPa. This implies that measured pore pressures exceeding 1 kPa absolute should theoretically compromise data reliability due to air intrusion. However, both Wittmann’s pioneering measurements and subsequent studies by Scott, Slowik, and Hammer consistently reported valid readings at 10–60 kPa (Table 1), substantially exceeding theoretical predictions. Notably, during field monitoring of airport runways, Slowik observed sustained hydraulic continuity at PWP up to 75 kPa without characteristic breakthrough pressure decay. He postulated that cement paste infiltration at the probe interface might alter effective pore geometry and create secondary menisci, consequently elevated the operational AEV [33]. This phenomenon demonstrates that the actual peak PWP measurement is governed by the smaller of the two values: the AEV of the probe or the AEV of the cement-based material. When the probe becomes fully embedded in cement-based material, the controlling parameter shifts from probe geometry to the matrix properties at the interface, depending on the maximum pore size of the surrounding cement-based materials. This mechanism may explain why the peak PWP measurement often exceeds theoretical AEVs calculated based on probe geometry. The resulting AEV depends critically on the factors like particle size distribution, water-to-binder ratio, and the permeability of the cement-based materials at the probe interface [31].

This mechanistic framework may elucidate Wittmann’s comparatively lower maximum PWP measurements (1976) obtained with analogous probes. Although his publication omitted cement fineness data, the characteristic particle size of circa-1976 cements substantially exceeded post-1990 formulations [56], potentially reducing the actual AEV. This interpretation aligns with Scott’s demonstration that maximum measurable PWP increases with decreasing water-to-binder ratios [31]. Consequently, when employing probes with intrinsically low AEVs, the following factors may influence the measured maximum PWP value, i.e., variations in cement particle sizes, incorporation of finer mineral admixtures (e.g., silica fume with particle diameters of 0.1–0.5 μm), variation in water-to-binder ratios, use of chemical admixtures, etc. Moreover, if the air entry value of the probe is governed by the cement-based material itself, the measured PWP peak value becomes sensitive to localized air infiltration processes within the pore network, potentially leading to significant spatial heterogeneity in the measurement results [35,57].

**Figure 5:**
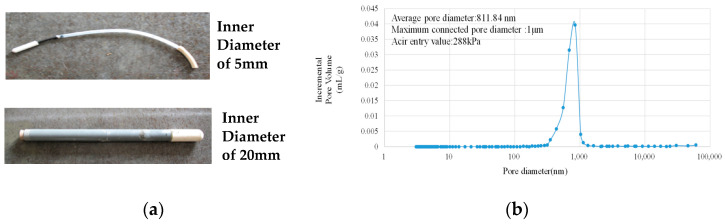
Ceramic probes and the pore size distribution according to mercury intrusion porosimetry (MIP): (**a**) Ceramic probes; (**b**) Pore size distribution by MIP.

**Figure 6:**
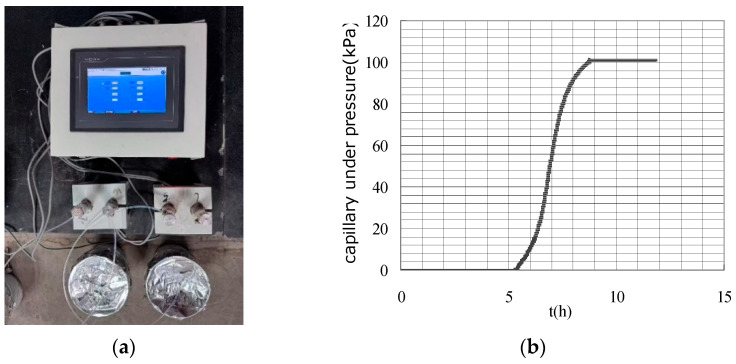
Testing setup and measuring result with ceramic probe: (**a**) Testing setup; (**b**) Measurement result [42].

(2) Tensiometers with ceramic probes. The engineered ceramic probe fabricated from high-temperature calcined kaolin exhibits a monolithic microstructure characterized by uniform micron-scale porosity (Figure 5). This controlled pore architecture enables air-entry values (AEV) exceeding 100 kPa, effectively eliminating air intrusion issues across high-pressure measurement regimes. Unlike conventional water-filled tube probes relying on singular orifices (1–3 mm diameter), this monolithic ceramic design provides omnidirectional pore distribution across the probe surface and order-of-magnitude increase in cement-probe interfacial contact area, ensuring stable hydraulic continuity through percolative pathways and enhanced measurement stability by mitigating localized failure points. Liu and Tian et al. began to use such kind of ceramic probes in studies on the mechanisms of autogenous and plastic shrinkage of cement-based materials [38,39]. As illustrated in Figure 6, they achieved sustained PWP measurements of 80–90 kPa without breakthrough decay, demonstrating reliable operation near the calculated theoretical capillary limit of tensiometers. The extended measurement range eliminates the influence of cement-based material properties on the measurement results within this measuring range. This advancement enables more precise tracking of the material’s behavior during the critical early stage from casting through initial hardening phase, till a couple of hours post-final set with eliminated sensitivity to cement-based materials properties. Subsequent studies by Sadegh and Pietro adopted identical probes for plastic shrinkage cracking investigations [49,50].

(3) High-capacity tensiometers with high-AEV ceramic probes. The use of high air-entry value (AEV) ceramic probes effectively eliminates external gas intrusion across extended measurement ranges. Yhe ultimate measurement limit is constrained by water cavitation within the internal hydraulic system. At ambient temperature, the relationship between the boiling point of water T (K) and negative pressure (below standard atmospheric pressure) can be described by the Clausius–Clapeyron equation, as follows:

(3) $$\ln\left(\frac{P}{P_{0}}\right)=-\frac{∆H_{vap}}{R}(\frac{1}{T}-\frac{1}{T_{0}})$$

where P_0_ is standard atmospheric pressure = 101.325 kPa; T_0_ is the boiling point at standard atmospheric pressure = 373.15 K (100 °C); ΔH_vap_ is the molar enthalpy of vaporization of water = 40.7 kJ/mol; R is the ideal gas constant = 8.314 J/(mol·K)

Consequently, when the gauge pressure measured by the tensiometer approaches −100 kPa (near the theoretical cavitation limit of −97.8 kPa at 20 °C), the absolute pressure within the water drops to the saturation vapor pressure. At this thermodynamic state, spontaneous vapor bubble nucleation occurs, triggering violent phase transition (cavitation). The rapid formation of vapor cavities disrupts hydraulic continuity, causing an abrupt decline in measured PWP. This fundamental physical limitation constrains the effective measurement range of tensiometers.

**Figure 7:**
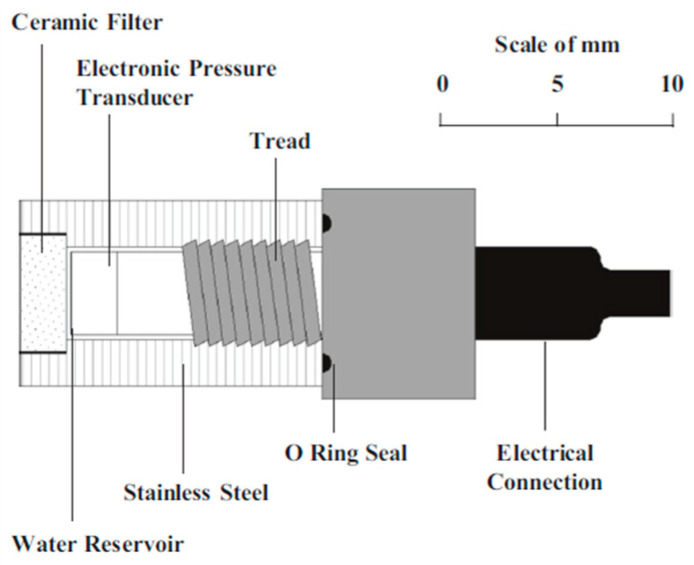
Design of HTC [58].

In soil research, to extend the measurement range of tensiometers, Ridley and Burland at Imperial College London developed the high-capacity tensiometer (HCT) in 1993. This instrument can directly measure negative pore water pressure (PWP) up to 1500 kPa in soils [58] (see Figure 7). The core components of the HCT comprise (1) a ceramic probe with an air entry value of 1500 kPa, (2) a miniature water reservoir with a volume of approximately 3 mm^3^, and (3) a pressure sensor compatible with the working pressure range corresponding to the probe’s air entry value. These components are all encapsulated within a stainless steel cylindrical container. The HCT’s measurement principle remains consistent with conventional tensiometers, relying on thermodynamic equilibrium between the water in the measured system and the probe’s internal water through a water-saturated porous ceramic. However, its key innovations include the use of a ceramic probe with a higher air entry value and a significantly smaller water-filled chamber above the probe.

By refining the maximum pore size of the ceramic probe, its air entry value can be increased to 15 Bar or higher. This enhancement prevents measurement errors caused by external gases entering the probe through the measured system during high-suction measurements. The adoption of a miniature water-filled chamber (volume: 3–5 mm^3^) aims to eliminate inaccuracies caused by water cavitation in the chamber under low hydraulic pressures, as discussed earlier. Research indicates that cavitation in capillary water requires overcoming additional energy barriers from surface tension and interfacial adsorption layers, resulting in significantly higher negative pressure thresholds for cavitation compared to water in pipes of ordinary diameters [59]. Therefore, in theory, a sufficiently small water-filled chamber requires greater energy for bubble formation, allowing the negative pressure threshold for cavitation to be markedly increased relative to conventional chambers. Consequently, most high-capacity tensiometers documented in the literature feature water reservoirs smaller than approximately 10 mm^3^. This underscores the critical need to minimize the water-filled chamber volume when designing high-capacity tensiometers intended to measure suction near the ceramic probe’s air entry value [60,61].

**Figure 8:**
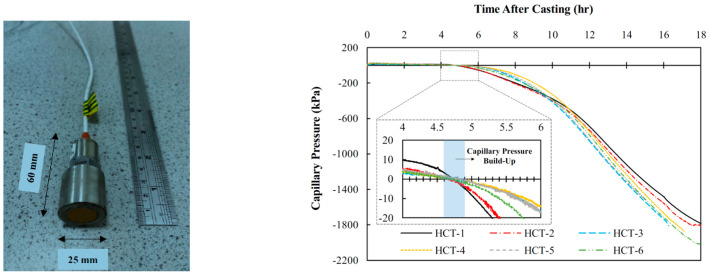
Probe and testing results of HTC-C [52].

Building on high-capacity tensiometers used in soil research, Jamali at Northumbria University modified the design to develop a high-capacity tensiometer for cement-based materials (HCT-C) [51,52,53], as illustrated in Figure 8. The HCT-C incorporates a thermally compensated pressure sensor to address temperature rises caused by rapid hydration in early-age cement-based materials, thereby eliminating temperature-induced measurement errors. This sensor, equipped with temperature compensation (16–71 °C), accurately records capillary pressure fluctuations in concrete under temperature variations induced by hydration or environmental factors. Utilizing this device, the researchers measured PWP in self-compacting concrete up to 1800–2500 kPa. This significantly extended the instrument’s measurement range to cover changes occurring during the plastic, setting, and hardened stages of concrete. Similarly, in South Africa, Renier et al. employed HCTs featuring ceramic probes with air entry values of 300 kPa and 1500 kPa, measuring maximum PWP in concrete up to 300 kPa [54,55].

As shown in the measurement results in Figure 8, the high-capacity tensiometer demonstrates the capability to measure PWP exceeding 100 kPa in cement-based materials, with good repeatability. The fundamental principle of the tensiometer method relies on water migration through the ceramic probe into the cement-based material driven by pressure differentials. However, at very high negative pressures, pore connectivity becomes significantly reduced. Soil physics research indicates that water movement in pores smaller than 1 μm is slow and difficult to drain [20], suggesting that equilibration under such conditions might require extended time. Nevertheless, high-capacity tensiometers, whether applied to soil or cement-based materials, typically exhibit rapid response times, achieving equilibration within minutes. This efficiency enables sensitive direct monitoring of internal PWP in cement-based materials.

### 3.3. Tensiometer Implementation

#### 3.3.1. Saturation and Calibration

Once embedded in cement-based materials, the probe can begin measuring capillary pressure upon establishing hydraulic continuity between the tensiometer’s internal reservoir and the material’s pore network. The sensor connects to a multichannel data acquisition system, enabling automated continuous monitoring of PWP evolution in early-age cement-based composites at programmed intervals (typically 10–60 s). Regardless of configuration, all operational protocols require rigorous pre-saturation procedures to prevent gas intrusion. The saturation process can be classified into atmospheric pressure saturation and high-pressure saturation.

**Figure 9:**
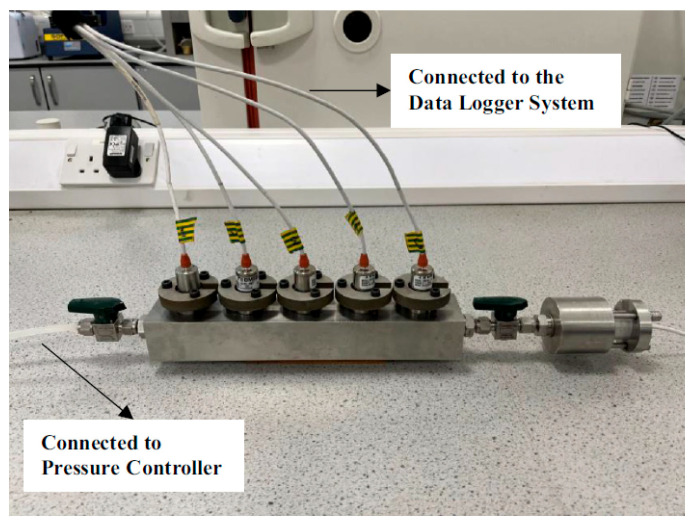
High-vacuum saturation of HCT-Cs [52].

(1) Atmospheric pressure saturation. For probes utilizing plastic hoses, injection needles, thin metal tubes, or ordinary ceramic tips, the saturation process is relatively straightforward and involves the following steps:
  - Prepare deaerated water. Boil tap water for at least 30 min to remove dissolved air, then cool it to room temperature;
  - Immerse and evacuate the probe. Soak the dry probe in the deaerated water for a minimum of 3 h while concurrently applying vacuum evacuation to remove residual gases.
(2) High-pressure saturation. Saturation protocols for high-capacity tensiometers (HCTs) utilizing high-AEV ceramic probes are significantly more complex due to the ceramic’s dense pore structure. Saturation is a critical prerequisite for proper HCT operation, as even minute air bubbles within the reservoir can lead to failure at pressures exceeding 100 kPa due to air compressibility. Consequently, a specialized high-pressure vacuum saturation procedure is essential. Figure 9 illustrates the high-vacuum saturation method for HCT-Cs introduced by Jamali [52]:
  - Initial Vacuum Treatment: Place completely dry HCT-Cs into a saturation chamber. Connect one end of the chamber to a Pfeiffer DUO 11 two-stage rotary vane vacuum pump (capable of achieving an absolute pressure of 3.10 × 10^−4^ kPa) and evacuate for 1 h to eliminate residual air.
  - Pressurized Water Infusion: Connect the opposite end of the chamber to a 4 MPa GDS Instruments pressure-volume controller. Apply 3000 kPa of pressure using deionized/deaerated water and maintain overnight (typically 12–16 h) to saturate the ceramic.
  - Post-Saturation Handling: Remove the HCT from the saturation chamber approximately 2 h before use. Immediately immerse it in deionized water to prevent cavitation, then reset the pressure readings to 0 kPa for calibration.

After saturation, the HCT-Cs were calibrated. The calibration of HCTs can be performed by cycling a positive pressure range using the pressure controller. Then, due to the symmetrical design and build of the pressure transducer, the measurements in the negative pressure range (capillary pressure) were successfully extrapolated from the obtained linear equation [62,63]. The calibration results showed perfect linearity through the collected points for both increasing and decreasing pressure, independent of the pressure transducer used in the assembly of the HCT-Cs.

#### 3.3.2. Orientation and Embedding Depth of Probes

**Figure 10:**
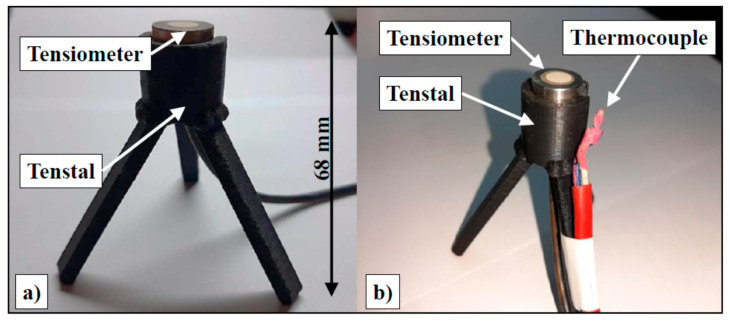
Three-dimensional printed pedestal for (**a**) tensiometer (**b**) tensiometer and thermocouple [54].

Due to varying experimental setups and research objectives, the orientation and embedding depth of probes differ significantly across the literature. Probe orientation primarily involves either horizontal or vertical placement. Inserting a probe vertically into cement-based material is relatively straightforward, with most probes oriented perpendicular to the surface. However, achieving consistent embedding depth can be challenging. Practitioners typically use a ruler to mark the target depth before slow insertion. The final position may deviate due to concrete density, workability, and probe weight. Deysel et al. addressed this by using a specialized 3D-printed pedestal, made in University of Pretoria in South Africa, to fix probe positions (Figure 10) [54].

**Figure 11:**
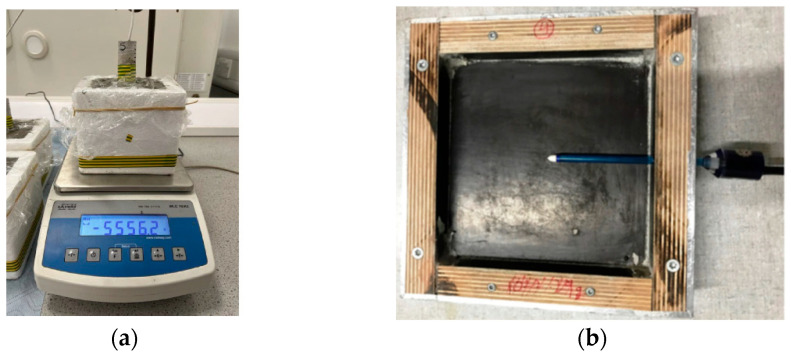
Emmbeded orientationof the probe: (**a**) Vertical [52]; (**b**) Horizontal [50].

Inserting probes horizontally requires passing them through the side of molds (Figure 11). This orientation often provides greater positional stability compared with vertical placement and is essential for measuring PWP gradients at different depths using ceramic probes. Wittmann, Slowik, and Ghourchian all employed horizontal probe placement in their studies [24,34,49]. Radocea suggests that the measured maximum capillary pressure depends on meniscus stability at the probe-cement-based material interface. Horizontal tube placement may lead to higher porosity beneath the tube due to internal water drainage, potentially affecting results [28]. Unlike point-contact probes (e.g., water-filled plastic hoses, injection needles, thin metal tubes), ceramic probes contact the material over a certain height. Consequently, their embedding depth represents a range rather than a single point.

Under sealed conditions, test results show no significant differences for sensors embedded at various depths. While under drying conditions, test results are typically influenced by embedding depth. However, under moderate evaporation rates using thinner samples, PWP values show negligible differences near the drying surface (within depths of 10–20 mm), indicating liquid phase continuity in this zone [47].

#### 3.3.3. Temperature Influence

In most of the laboratory research, tensiometers have been used under room temperatures. Tian et al. studied the setting time of cement-based materials in the range of 10–36 °C, using tensiometers with ceramic probes [45]. Jamali et al. examined the impact of temperature on measurement accuracy in high-capacity tensiometers (HCTs), specifically addressing the rapid temperature rise occurring during cement hydration [52]. Their research revealed that even systems equipped with thermally compensated pressure transducers exhibit temperature-dependent variations in pressure readings. The magnitude of this effect shows a direct correlation with heating rates; faster temperature increases produce larger deviations in pressure measurements. However, under typical operating conditions where temperature changes in cement-based materials remain moderate (both in rate and magnitude), the influence on pressure readings is insignificant. A more comprehensive study about the influence of temperature in this method is still needed in the future.

#### 3.3.4. Field Adaptability

In addition to laboratory research, researchers have successfully applied tensiometers in field engineering applications. However, due to site constraints, implementing the high-pressure saturation procedure for HCTs may prove difficult, potentially hindering the field deployment of such instruments. While tensiometers with tube probe and ceramic probes using the atmospheric saturation procedure have demonstrated reliable performance at construction sites [33,43], field experience has revealed limitations with wired configurations; cables interfere with surface finishing operations and are vulnerable to disruption of the hydraulic connection between pore water and sensors during movement. Implementing multi-point measurements on large-scale structures like concrete pavements or bridges presents additional challenges.

To address these limitations, Slowik developed a wireless pore water pressure (PWP) sensor by integrating a radio communication module into an existing wired system [33]. The device automatically connects to a base station within a range of approximately 60 m. It features a fully sealed, IP-rated enclosure, with only the probe interface and antenna port exposed. Charging is enabled via electromagnetic induction using a magnetic switch, allowing for non-contact operation. This innovation facilitates more convenient and reliable in-situ PWP measurement in cement-based materials.

Field measurements using this wireless capillary pressure sensor have been conducted at over ten construction sites, including airfield and roadway projects. An enhanced version of the sensor, featuring extended radio range, is currently under development. Beyond measuring capillary pressure, this upgraded device will also monitor concrete temperature and key environmental parameters such as air temperature, relative humidity, wind speed, and solar radiation. The integration of these parameters will enable estimation of the evaporation rate, bleeding water volume, and near-surface concrete maturity. However, the associated cost of this integrated instrumentation requires careful evaluation for its future practical application. There is no recommendation about practical PWP methods for various applications yet; more experience and testing may be needed in the future.

In summary, after decades of development, researchers have significantly enhanced the measurement range and reliability of instruments for PWP monitoring in cement-based materials. Devices using water-filled plastic hoses, injection needles, or thin metal tubes as probes offer structural simplicity and ease of use. However, their larger open-tip diameters and single-point hydraulic contact result in a limited measurement range, with recorded maximum PWP values dependent on material properties and probe placement. The introduction of sintered ceramic probes—featuring uniformly distributed micropores—significantly improves performance. Their densified pore structure increases the air-entry value (AEV), while the large-area hydraulic contact with cement-based materials extends both the measurement range and reliability. This advancement enables PWP monitoring not only during the plastic stage but also throughout the plastic-to-setting transition. Furthermore, the operational simplicity and cost-effectiveness of these ceramic-probe devices facilitate practical field deployment. High-capacity tensiometers (HCTs) address remaining limitations by further extending the PWP measurement range up to 2500 kPa. This is achieved through refined ceramic probes and specially designed miniaturized water reservoirs, allowing measurements during early hardening stages and bridging the gap between conventional tensiometers and traditional hygrometers. However, the complex high-pressure pre-saturation procedures required for HCTs may impede their adoption in field engineering applications. There is currently no direct comparison between different testing instruments, and data on the test accuracy and reproducibility of each instrument and method are still lacking. Future studies are needed to establish standard unified methods.

## 4. Application of Early-Age PWP Testing Methods in Cement-Based Materials

Tensiometers are uniquely valuable in early-age cement-based material studies, enabling continuous monitoring and real-time tracking of internal moisture’s energy state evolution as hydration proceeds. The major applications cover ordinary Portland cement concrete, self-compacting concrete, shortcrete, and 3D-printed concrete under evaporation exposure as well as sealed conditions.

### 4.1. Investigating the Mechanism and Mitigation of Plastic Shrinkage Cracking in Cement-Based Materials

Freshly placed cement concrete exposed to drying conditions is susceptible to plastic shrinkage cracking. As this occurs before concrete setting while cement hydration effects remain negligible, the primary driven mechanism of cracking is water evaporation. Early research frameworks consequently focused on evaporation analysis to explain plastic cracking mechanisms, as seen in standards such as ACI 305.1-06 [64]. These studies developed an evaporation nomograph through extensive experimentation, integrating concrete surface parameters (temperature, vapor pressure) with environmental factors (ambient temperature, humidity, wind velocity, and solar radiation). Jensen [65] invented a curing meter to directly measure concrete evaporation rates using thermodynamic principles, thereby overcoming operational complexities associated with nomograph calculations. However, significant variations exist in critical evaporation rate thresholds across different mix designs. For example, silica fume additions substantially reduce these thresholds, challenging the universal applicability of evaporation-based control methods.

Measuring PWP provides direct characterization of the driving force behind plastic shrinkage cracking. The pioneering work by Wittmann [24], who first monitored PWP in cement-based materials, fundamentally aimed to investigate plastic shrinkage mechanisms. Radocea [26,27] employed similar apparatus to establish the relationship between PWP and water evaporation, subsequently proposing predictive models for plastic shrinkage. Based on PWP monitoring results, Liu and Tian et al. [44] developed a two-stage plastic shrinkage model. It is widely accepted that PWP development integrates the cumulative effects of environmental conditions, specimen geometry, and material composition on plastic shrinkage. Prior to negative PWP development (dP/dt = 0), shrinkage manifests as vertical settlement quantitatively equivalent to evaporated water volume. Once negative PWP initiates (dP/dt > 0), horizontal shrinkage commences and increases linearly with PWP magnitude. When evaporation-induced PWP reaches a critical value, air penetrates through the largest surface-connected pores. This triggers moisture redistribution within the cementitious matrix, with the corresponding PWP representing the material’s air entry value. Crucially, PWP measurements continue to increase provided either the material’s air entry value remains lower than the tensiometer probe’s threshold, or air has not penetrated the probe itself [34].

The growth of PWP provides the direct driving force for plastic shrinkage cracking. During this stage, hydration processes are typically negligible, and the evolution of structural resistance is governed by PWP development. Surface moisture evaporation generates suction pressure in cement-based materials, forming a stiffened surface layer (“hard skin”) with low tensile strain capacity. As evaporation progresses, this layer propagates deeper into the material, in a phenomenon that is quantifiable through multi-depth PWP measurements under drying conditions [23]. The formation of this layer further demonstrates the correlation between plastic tensile strength and PWP. Wang et al. [46] established quantitative relationships between PWP and key mechanical properties. Within their tested PWP range (~70 kPa), plastic tensile strength increased rapidly with rising PWP before stabilizing at higher pressures. This relationship remained consistent across material compositions and mix designs. Similarly, the plastic-stage elastic modulus exhibited an approximately linear relationship with PWP. Critically, PWP correlates systematically with plastic shrinkage, elastic modulus, and tensile strength independently of material variables, providing a robust basis for cracking prediction. Slowik’s research [34] demonstrates that when evaporation exceeds bleeding, depletion of water in surface-connected pores weakens interparticle bonds, triggering system instability and cracking risk. Air entry corresponds to three phenomena, as follows: (1) settlement rate maximum, (2) onset of deviation between specimen volume change and evaporated water volume, and (3) abrupt electrical conductivity reduction. Measured air entry values range from 10–48 kPa. Liu et al. [47] determined a critical cracking PWP of 20–30 kPa for cement paste through theoretical and experimental analysis. Ghourchian et al. [49] developed a poromechanical model to analyze the plastic shrinkage cracking mechanism of cement mortar, indicating that when cracking occurs and the system remains nearly saturated, the increase in PWP leads to a simultaneous increase in material stiffness as well as plastic shrinkage, which may lead to cracking when the shrinkage stress exceeds the critical value of tensile strength. The bulk modulus evolution plays a prominent role in controlling the plastic shrinkage of cement-based materials. Their model suggests a safe PWP threshold of 3–4 kPa for crack mitigation.

**Figure 12:**
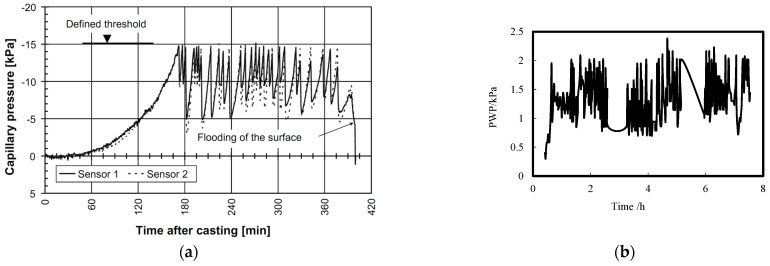
Close-loop rewetting curing method based on in situ PWP monitoring: (**a**) Defined threshold of 15 kPa [34]; (**b**) Defined threshold of 2 kPa [43].

Despite variations in proposed safe PWP thresholds for mitigating plastic shrinkage cracking across literature, a consensus recognizes that monitoring and controlling in-situ PWP within established limits can prevent such cracking. Slowik et al. [33] employed the material’s air entry value as the PWP threshold, developing a closed-loop rewetting system for fresh concrete surfaces using real-time PWP monitoring (Figure 12a). Implemented on airport runways and highway pavements, this method significantly reduced early-age cracking risk. Tian and Wang et al. [42,66] proposed an intelligent initial fogging technique maintaining surface PWP within a 2 kPa threshold through continuous monitoring, effectively preventing cracking in both ordinary (C35) and ultra-high-performance concrete (Figure 12b). Deysel et al. [54,55] introduced a No-Cracking Capillary Pressure Boundary Model, calculating mixture-specific critical pressure limits relative to evaporation rates. Their system utilizes live capillary pressure measurements to dynamically control pressure responses and prevent cracking across diverse evaporation conditions. Sayahi et al. [57] defined the critical period (t_cr_) as the interval between drying onset (t_d_, when PWP initiates) and initial setting (t_i_). Extended critical periods correlate with higher stress-to-strength degradation and cracking risk. They established a predictive model for cracking severity based on the area under the PWP curve during t_cr_, enabling cracking control through concurrent PWP and hydration monitoring.

Compared with evaporation-based cracking control methods, PWP enables remote in-situ monitoring at construction sites. This approach inherently integrates the combined effects of environmental conditions and material properties, facilitating more automated and reliable control of plastic shrinkage cracking risk.

### 4.2. Characterization of Early-Age Self-Desiccation Effects and Structural Formation of Cement-Based Materials

Self-desiccation shrinkage refers to the contraction occurring in sealed, isothermal-cured concrete after initial structural formation, where continued cement hydration generates empty pores that reduce internal relative humidity (IRH) [57]. Under restrained conditions, the superposition of self-desiccation-induced shrinkage stress and thermal contraction stress induces a much higher early-age cracking risk in low water-binder ratio concrete than in traditional concrete. The IRH reduction caused by water consumption during hydration is termed as the self-desiccation effect, which is generally recognized as the direct driven mechanism of self-desiccation shrinkage (autogenous shrinkage after “time zero”) despite the disputations between the proposed theories. Hygrometry is a conventional method for RH measurement and is widely adopted in cement concrete research field. Previous studies have characterized a quantitative relationship between IRH and self-desiccation shrinkage based on the measurement results of hygrometers [11,67]. However, it should be noted that a hygrometer can measure RH in only the hygroscopic range (normally up to approximately 98% at most). This is insufficient for very early-age concrete because the materials are still in the superhygroscopic range (approximately in the range of 98–100%). Another drawback of hygrometers is the long balance time with the measured cement or concrete (around 10–12 h or more), which also prohibits the investigation of RH changing at the beginning of desiccation (drying or self-desiccation) [20]. Furthermore, the relative humidity measured by a humidity meter includes a decrease caused by dissolved salt ions in pore solutions. To calculate the true critical radius for use in autogenous shrinkage modeling, it is necessary to test different hydration ages and metal ion concentrations before applying the Rasulf equation to determine the relative humidity increase due to hydration, and subtract this from the measured value.

The use of tensiometers in cement-based materials overcomes the abovementioned limitations of hygrometers, moving the starting time of IRH measurement to the finish of placement and compensating for the information loss with conventional hygrometers. By continuous monitoring of PWP of cement-based materials from the finish of placement, we can calculate the corresponding IRH based on Laplace’s formula and Kelvin’s law, as follows:

(4) $$RH=\frac{P_{g}}{P_{sat}}=exp\left(-\frac{2\gamma M_{l}cos\theta }{r\rho_{l}RT}\right)=exp\left(-∆P\cdot M_{l}cos\theta /(\rho_{l}RT)\right)$$

where *RH* represents relative humidity, Δ*P* denotes the pore negative pressure, *P_sat_* signifies the saturated vapor pressure of plane water, *P_g_* indicates the saturated vapor pressure of surface water, *M* is the molar mass of the liquid phase, *R* is the ideal gas constant, *T* denotes the absolute temperature, and *ρ_l_* represents the density of the liquid phase.

**Figure 13:**
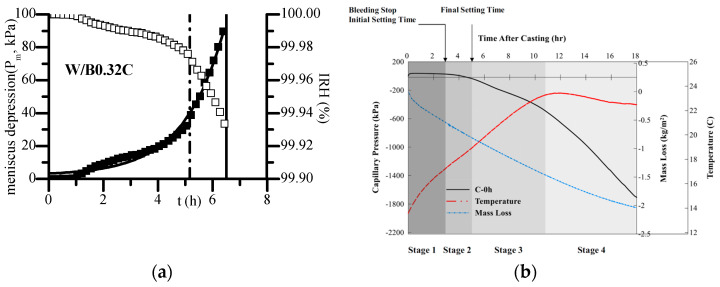
IRH measuring with tensiometer for early-age cement-based materials: (**a**) Ordinary tensiometer [39]; (**b**) High-capacity tensiometer [53].

Given that PWP measuring with tensiometers is not influenced by salt dissolution, the calculated IRH can be directly related to self-desiccation shrinkage. Radocea [27] proposed an autogenous model based on the measurement of early-age PWP, concerning the vertical and horizontal deformation of a plastic or semiplastic concrete mass (i.e., when the dead weight of concrete cannot be neglected). Tian et al. [38,39,40,41] employed this method to investigate early-age self-desiccation for cement paste with a water-to-cement ratio of 0.32. As illustrated in Figure 13a, although the calculated self-desiccation effect is minor, it should be noticed that there is a distinct transition point in the development curve of IRH at early-age. Prior to this point, the changes in PWP and IRH are both negligible, and IRH remains approximately 100%; after this point, PWP increases rapidly as hydration progresses, leading to a swift decline in IRH. Within hours, PWP quickly surpasses 85 kPa, with a corresponding Kelvin critical radius around 1~2 μm and an IRH of about 99.9%. Despite the material still being close to saturation, there is a noticeable self-desiccation effect occurring near the hardening stage. Such accordingly minor shrinkage stress might lead to rapid shrinkage development if the concrete still has large deformation capacity. Based on this method, researchers have also investigated the effect of water-to-binder ratio, mineral admixtures, water-reducing agents, and bleeding on early self-desiccation shrinkage of cement-based materials. By means of the developed high-capacity tensiometers, Armin [51] et al. studied the PWP development of early-age self-compacting concrete till the hardening stage, under standard environmental conditions (temperature: 20 ± 2 °C; relative humidity: 60 ± 5%; moderate wind speed). Although their measured PWP included the combined relative humidity decline due to water evaporation as well as self-desiccation effects, it was observed that after final setting, self-desiccation effects gradually took over as the dominant factor (see Figure 13b). The measured values of PWP were between 1800–2500 kPa at the end of testing, corresponding to approximately 98% relative humidity. This measurement period aligned well with the deceleration phase of hydration (age of 1d), providing a good correlation with hygrometer measurements, which may help in further study of the self-desiccation effect of cement-based materials through the whole course of hydration.

**Figure 14:**
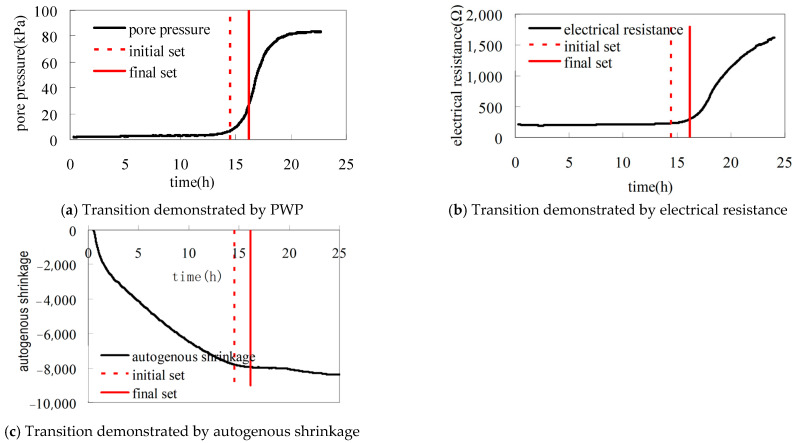
Correlation of the transitions in the curve of PWP (**a**), electrical resistance (**b**) and autogenous shrinkage (**c**) of early-age cement paste [42].

The early hydration of cement-based materials undergoes a critical transition from fluid to solid state. This period features discontinuous changes in physical properties, with structural skeleton formation holding particular significance, notably for determining ‘time-zero’ (the reference point for self-desiccation shrinkage [SDS] measurement). Premature SDS measurement initiation (before ‘time-zero’) yields meaningless data, while delayed initiation underestimates shrinkage magnitude and thus cracking risk. There are no standardized criteria for the termination of “time-zero” in self-desiccation. PWP monitoring offers a viable approach to establish ‘time-zero’ based on its physical definition. Determination of ‘time-zero’ through PWP curve transitions shows excellent agreement with methods using electrical resistance or autogenous shrinkage transitions (Figure 14) [42]. Further studies are still needed to provide comparison with other kinds of methods.

Hammer [23] identified PWP as an effective tool for monitoring the evolution of physical properties in fresh cementitious systems (paste, mortar, or concrete) during setting. Based on PWP measurements, the process is divided into two distinct stages relative to the point of self-support (PSS). Under sealed conditions, before PSS, PWP corresponds to the hydrostatic pressure generated by cement-based materials, which is determined by the height over the point of measuring and the density. As a “self-supporting” structure forms, pressure decreases over time. When materials can support their own weight, PWP equals water pressure at the measurement depth. Changes in PWP during this stage can be used to evaluate workability retention, period of consolidation, and period of formwork pressure. In the period after PSS, under sealed conditions, PWP reflects quite well the stiffness evolution in paste–mortar–concrete systems. The PWP increases at a low rate until the period when setting starts and then increases at a higher rate. The time when PWP reaches this high rate coincides fairly well with the time of setting. The friction force in slip-form construction is also mainly caused by the tension of PWP in the time until setting. PWP can serve as an effective indicator for friction in slip-form construction.

**Figure 15:**
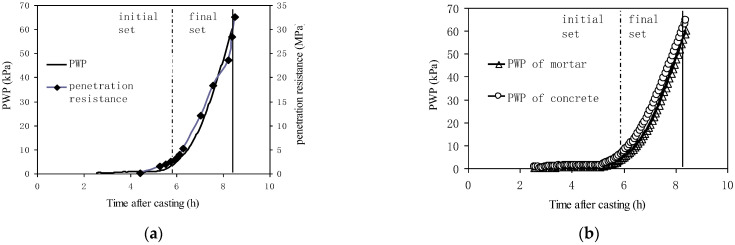
Monitoring of initial setting of concrete based on pore water under pressure measurement [42]. (**a**) Relationship between PWP and penetration resistance development. (**b**) PWP development of mortar and concrete.

Tian et al. [42,45] established the relationship between PWP and penetration resistance in sealed fresh concrete with diverse compositions. Their experimental results (Figure 15) demonstrate that, when bleeding water is eliminated, there was a high similarity in the development curves of these two parameters, regardless of variations in temperature, raw materials, and mix proportions. Moreover, the PWP development curves of the concrete and the remaining mortar after sieving out the coarse aggregates (particle size > 5 mm) were also identical. The water adsorption of coarse aggregate was not discussed in detail; its possible influence may need further investigation. Based on these experimental results, a PWP-based method for determining setting time was proposed. These findings enabled a novel PWP-based method for setting time determination. By converting penetration resistance data into equivalent PWP measurements, this approach facilitates remote, continuous monitoring and automated in situ tracking of the setting process for structural concrete at construction sites.

### 4.3. Application of PWP Measurement in Novel and Special Concrete

Beyond applications in conventional Portland cement concrete, pore water pressure (PWP) measurements have recently been extended to novel concrete types, such as shotcrete and 3D-printed concrete. Patent CN 116429818 A describes a method for determining setting time using a tensiometer with a ceramic head. In this method, a ceramic probe equipped with a pressure transducer is affixed to reinforcement steel prior to concrete spraying. Preliminary experimental results demonstrate that the transition point in the PWP evolution curve coincides well with the hydration heat peak in shotcrete mixes (water-to-binder ratio = 0.35–0.50, accelerator content = 2–10%) [68].

Ghourchian et al. [69] measured pore pressure development in 3D-printed concrete using tensiometers with a maximum capacity of ~80 kPa (a typical air-entry value, AEV, for ceramic probes). Their results indicated that 3D-printed materials exhibited a faster increase in capillary pressure compared with cast concrete. Furthermore, partial substitution of cement with silica fume was found to further accelerate capillary pressure development.

While this method is well established for conventional concrete, its application to these novel and special concretes remains limited to date. However, its potential is significant and likely to attract increasing research interest in the future.

## 5. Conclusions and Outlooks

The evolution of early-age performance in cement-based materials is intrinsically linked to changes in internal water potential, which directly represent the Gibbs free energy of water within the material. The adaptation of soil science tensiometers enables automated, remote, real-time, in situ monitoring of this water potential and its evolution. This methodology offers distinct advantages for both fundamental research and practical engineering applications concerning early-age material properties. With the development of AI in the concrete industry, the integration of this measuring technique with BIM, AI, and digital twins may provide promise for quality control and service-life enhancement for early-age cement-based materials.

- While sharing common measurement principles, tensiometer probe designs vary significantly across studies, leading to differences in measurement range and operational characteristics among different tensiometers. Future comparative studies among different laboratories are needed to further evaluate the repeatability and reproducibility of test results and establish a standardized methodology for this approach. Future standardization efforts must also address application-specific requirements while balancing measurement capabilities with operational feasibility.
- Under drying conditions, PWP in fresh cement-based materials demonstrates robust correlations with plastic shrinkage, tensile strength, and elastic modulus. These relationships persist across diverse material compositions and environmental conditions, establishing PWP as a reliable predictor of plastic shrinkage cracking. While reported PWP cracking thresholds vary in the literature, consensus confirms that real-time monitoring of PWP at construction sites and maintaining it within defined thresholds can effectively prevent cracking. This forms the basis for closed-loop automated fog-curing systems utilizing in-situ PWP monitoring, which have proven successful in mitigating such cracking in high-performance cement-based materials during field implementation.
- Under sealed conditions, PWP measurement provides direct and sensitive monitoring of initial self-desiccation in cement-based materials from the moment of water addition. This approach overcomes the limitations of hygrometer methods during high-humidity stages. The measured PWP curves exhibited a distinct transition period demonstrating strong correlations with other kinds of signals like penetration resistance, electrical resistance and autogenous shrinkage etc. These correlations, combined with the capability for remote, real-time, automated, and in situ PWP monitoring, create significant potential for characterizing critical parameters, including setting time, slump retention, bleeding, slip-form friction, etc., in both laboratory and construction sites.

## Figures and Tables

**Table 1:** Details of testing instrumentation and methodologies in research.

Research Team	Research Subject	Sensor Type	Probe Orientation	Embedment Depth	Saturation Method	Peak PWP
Wittmann, 1976 [24]	Plastic shrinkage	Plastic tube with a paper stopper	horizontal	7.5 cm	De-aired water saturation	10~20 kPa
Radocea, 1991~1998 [25,26,27,28]	Plastic shrinkage, autogenous shrinkage and bleeding	3 mm inner-diameter tube	vertical	10~60 mm	De-aired water saturation	60~80 kPa
Scott et al., 1997 [31]	Early-age shrinkage cracking	5 mL syringe	vertical	1.5 cm	De-aired water saturation	~30 kPa
Hammer, 2001~2006 [23,29,30]	Early-age shrinkage cracking	3 mm inner-diameter steel tube	vertical	5 mm, 50 mm	De-aired water saturation	~20 kPa
Liu et al., 2006~2024 [38,39,40,41,42,43,44,45,46,47,48]	Early-age shrinkage cracking	calcined porous ceramic probe	horizontal & vertical	5~50 mm	De-aired water saturation	80~90 kPa
Holt et al., 2001, 2004 [36,37]	Early-age shrinkage cracking	Type KP-2A	vertical	3 cm, 6 cm	De-aired water saturation	40~60 kPa
Slowik et al., 2008~2013 [33,34,35]	Plastic shrinkage cracking	3 mm inner-diameter brass tube	horizontal	4 cm	De-aired water saturation	60~80 kPa (15 kPa for concrete)
Ghourchian et al., 2019 [49,50]	Plastic shrinkage	200 kPa AEV porous ceramic probe	horizontal	1 cm, 8.5 cm	De-aired water saturation	~80 kPa
Jamali et al., 2022~2024 [52,53]	Early-age shrinkage	1500~2000 kPa AEV HCT-C	vertical	25~75 mm	High-pressure saturation with deionized water	1800~2500 kPa
Deysel 2022~2023 [54,55]	Plastic shrinkage cracking	300 kPa~1500 kPa AEV HCT	vertical	32 mm	High-pressure saturation with deionized water	~300 kPa

Note: In Table 1, AEV represents the air entry value in Equation (2), as demonstrated in Section 3.2.

## Data Availability

No new data were created or analyzed in this study. Data sharing is not applicable to this article.
